# PROVISION OF SOCIAL WORK SERVICES DURING HOME HEALTH CARE: INDIVIDUAL- AND AGENCY-LEVEL DETERMINANTS OF ACCESS

**DOI:** 10.1093/geroni/igae098.2059

**Published:** 2024-12-31

**Authors:** Julia Burgdorf, Yolanda Barron-Vaya, Jeri Goodman, Margaret McDonald

**Affiliations:** VNS Health, New York, New York, United States; VNS Health, New York, New York, United States; VNS Health, New York, New York, United States; Visiting Nurse Service of New York, New York, New York, United States

## Abstract

Medicare-funded Home Health (HH) delivers skilled health care services via visits to patients’ homes. Social work (SW) services are included within the HH benefit, and qualitative research suggests SW positively impacts outcomes for HH patients with complex needs. However, no prior work has quantitatively assessed SW provision during HH. We examined 2018 linked HH claims, assessment, and HH agency data for a national sample of 1,372,563 Medicare HH patients. Just 11.3% of HH patients received SW; those who received SW had a mean of 1.3 (SD=0.6) SW visits and a mean of 8.8 days (SD=6.6) from HH admission until the first SW visit. We modeled odds of receiving SW using a multilevel logistic model clustering at the HH agency level and adjusting for patient sociodemographic characteristics and clinical status and HH agency characteristics. The patient-level factors most significantly associated with receiving SW included being Black compared to White (aOR: 1.66; 95% CI: 1.62-1.71), being Medicaid-enrolled (aOR: 1.24; 95% CI: 1.19-1.30), having no family caregiver (aOR: 3.83; 95% CI: 3.62-4.07), and having depression (aOR: 2.26; 95% CI: 2.20-2.33). HH agency-level factors associated with receiving SW were non-profit ownership (aOR: 1.40; 95% CI: 1.29-1.52), urban service area (aOR: 1.48; 95% CI: 1.33-1.64), 60 or more full-time employees (aOR: 1.89; 95% CI: 1.70-2.09), and having social workers on staff, compared to providing SW via contract (aOR:2.23; 95% CI: 2.05-2.41). Findings indicate that SW access remains rare during HH and is driven by both demand-side (i.e., patient needs) and supply-side (i.e., agency capacity) factors.

